# Bioactive Compounds and Antioxidant Properties with Involved Mechanisms of *Eugenia involucrata* DC Fruits

**DOI:** 10.3390/antiox11091769

**Published:** 2022-09-07

**Authors:** Giuseppe Mannino, Graziella Serio, Alberto Asteggiano, Noemi Gatti, Cinzia M. Bertea, Claudio Medana, Carla Gentile

**Affiliations:** 1 Department of Life Sciences and Systems Biology, University of Turin, Via Gioacchino Quarello 15/A, 10135 Turin, Italy; 2 Department of Biological, Chemical and Pharmaceutical Sciences and Technologies (STEBICEF), University of Palermo, Viale delle Scienze, 90128 Palermo, Italy; 3 Department of Molecular Biotechnology and Health Sciences, University of Torino, Via Pietro Giuria 5, 10125 Torino, Italy

**Keywords:** ABTS, CAA, gene expression, antioxidant enzymes, oxidative stress, anthocyanins, HPLC-Orbitrap, Cereja-do-Rio-Grande

## Abstract

In this study, the phytochemical profile and the antioxidative properties of *Eugenia involucrata* fruits were evaluated. Spectrophotometric assays indicated that these berries are a rich source of polyphenols with very high radical-scavenging and metal-reducing activities. High-performance liquid chromatography–Orbitrap analysis was able to carry out the annotation of 36 different compounds, mainly belonging to the flavonol, flavan-3-ol, and anthocyanin families. Antioxidant activity of the fruit extract was evaluated in a cell-based lipid peroxidation model. Obtained data showed that the extract, at very low concentration, was able to prevent oxidative damage in HepG2 cells exposed to oxidative stimuli. Moreover, the evaluation of the gene expression of the most important antioxidant enzymes suggested that the observed antioxidant protection in cells also involves an improvement in enzymatic antioxidant defenses. Finally, the collected data show that E. involucrata fruits are a good source of natural antioxidant molecules and provide evidence of their potential application in the nutraceutical field.

## 1. Introduction

The climate change affecting our planet in the last century has strongly impacted crop management in different countries. These changes have shifted several crops toward higher elevations and latitudes, with a medium rate of 11.0 m and 16.9 km per decade, respectively [1], and have promoted more resilient plant species. For example, in sub-Saharan Africa, the area suitable for the cultivation of maize and beans has shrunk by about 60 per cent to make space for more tolerant crops, such as sorghum and millet [2]. The same phenomenon has also been observed in other countries, including those in North America, South America, and several Mediterranean areas, such as Spain, Portugal, and Italy [3,4]. On the other hand, climate change has allowed for the introduction of non-native plants in geographical areas where suitable environmental conditions have been naturally established [5]. Consequently, a diversification of agricultural production with positive effects on biodiversity and ecosystem and a reduced loss in economic profit have been achieved.

Sicily is the largest island of Italy, located in the middle of the Mediterranean Sea. In this geographical area, the climate changes of the last century have led to hot and humid summers, mild winters, and very temperamental middle seasons. These particular climatic conditions, very similar to the tropical ones, have made Sicily well-suited to the cultivation of non-native tropical plants, such as kiwi, papaya, avocado, litchi, and mango [6]. Consequently, Sicily, which was previously known almost exclusively for producing oranges and lemons, is also becoming a producer of exotic fruits. Furthermore, the experimental cultivation of other tropical species, including jaboticaba, passion fruit, star fruit, and black sapote has recently been successfully investigated [6].

Recent studies evaluating the quality traits of non-native plants cultivated in Sicilian territory have demonstrated that nutritional, nutraceutical, and sensorial traits were comparable, if not improved, with respect to fruits of plants cultivated in tropical native countries [6,7]. *Eugenia involucrata* DC is a native tree species from southern Brazil, belonging to the Myrtaceae family and distributed in tropical and subtropical regions. The 5650 species of Myrtaceae are organized in 150 different genera, among which *Eugenia* genus stands out as one of the greatest economic importance along with *Plinia* and *Myrciaria* (Souza et al., 2018). While most of *Eugenia* species are ornamental, others, such as *E. caryophyllata* (cloves), find interesting applications as flavoring in the cosmetic industry, or as a remedy in traditional medicine [8]. Moreover, some *Eugenia* species produce edible fruits that are almost exclusively known locally (i.e., Pitanga or Cereza do Brasil from *E. brasilensis,* and Grumichama or Cereza do Vayena from *E. uniflora*). Concerning *E. involucrata*, although it is mainly used as an ornamental plant, it is able to produce small edible fruits tasting like sweet cherry. The cherry, commonly known as Cerejeira, Cerella, or Cereza do Rio Grande, has a red-to-deep-purple color and a diameter of about 2.5 cm. Locally, the fruit is eaten fresh or used for jam or juice preparation [8]. Some recent works have reported how this cherry is a source of antioxidant bioactive compounds [8,9,10]. However, a complete phytochemical profile was not currently characterized, and the functional properties need further investigation. Finally, studies concerning *E. involucrata* fruits obtained in the Mediterranean environment are not present in the scientific literature.

This study aimed to characterize the phytochemical profile and antioxidant properties of *E. involucrata* fruits from plants grown in Sicily. The main classes of polyphenolic compounds were measured via spectrophotometric assays and the phytochemical profile was explored via HPLC-Orbitrap. The antioxidant properties were estimated in a cell model and the mechanisms involved in the observed effects were investigated, including the impact on enzymatic antioxidant defenses.

## 2. Materials and Methods

### 2.1. Standards and Chemicals

2,2′-azobis(2-methylpropionamidine)-dihydrochloride (ABAP), [2,2′-azinobis(3-ethylbenzothiazoline-6-sulfonic acid)]-diammonium salt (ABTS), 2′,7′-dichlorofluorescin diacetate (DCFH-DA), Folin–Ciocalteu reagent, Hanks’ balanced salt solution (HBSS), sodium carbonate (Na_2_CO_3_), dimethylacetamide (DMAC), proanthocyanidin A2-type (PAC-A2), 6-hydroxy-2,5,7,8-tetramethylchroman-2-carboxylic acid (Trolox), gallic acid (GA), potassium chloride (KCl), sodium acetate (NaCH_3_COO), potassium persulfate (K_2_S_2_O_8_), 2,4,6-tripyridyl-S-triazine (TPTZ), and iron chloride hexahydrate (FeCl_3_ x 6H_2_O), were purchased from VWR International (Radnor, PA, USA). Roswell Park Memorial Institute (RPMI) 1640 cell-culture medium, fetal bovine serum (FBS), phosphate-buffered saline (PBS), 200 mM L-glutamine solution, 170,000 U/L trypsin solution supplemented with 0.2 g/L of ethylenediaminetetraacetic acid (EDTA), and 10 mg/mL penicillin/streptomycin solution were purchased from Lonza (Verviers, Belgium). All other employed materials and solvents were of analytical grade unless otherwise indicated, and purchased from Sigma-Aldrich (St. Louis, MO, USA).

### 2.2. Plant Material and Fruit Extract Preparation

*Eugenia involucrata* DC. (Backer) was harvested from trees taxonomically identified by Giancarlo Torre (botanist) and grown in Vivai Torre s.r.l. (Milazzo, Sicily, Italy; 38°19′ N, 15°24′ E; 20 m above sea level). The ripe fruits were harvested, transported in refrigerated boxes, frozen in liquid nitrogen, and stored at −80 °C until extract preparation.

For extract preparation, the seed was separated from pulp and peel. The edible portion (pulp and peel) was finely chopped and carefully homogenized (VEVOR FSH2 1000 mL Homogenizer, Vevor Italia, Italy). Subsequently, hydroalcoholic extracts were freshly made as described in our previous publication [6]. Briefly, twenty grams of homogenate were weighted and extracted in 100% EtOH using a 1:10 (*w*/*v*) ratio. Samples were mixed by vortexing for 5 min at room temperature (RT), sonicated for 15 min at RT, and then centrifuged (30 min at 8000× *g*, 4 °C). To carry out an exhaustive extraction, the centrifugation residue was extracted twice using the same solvent and following the same procedure. Finally, supernatants were combined, filtered, concentrated in rotavapor (20 °C and 284 mmHg for 30 min) and stored at −20 °C until analysis had been carried out. The extraction procedure was repeated to obtain three different replicates.

### 2.3. Phytochemical Profile

#### 2.3.1. Total Polyphenol Content

The total polyphenol content (TPC) was measured through the Folin–Ciocalteu assay by adapting the protocol for a spectrophotometric reading using a microplate reader (Microplate Reader NeoReader^®^) [11]. Briefly, 6 µL of the mixture composed of phosphotungstic and phosphomolybdic acids was incubated along with 10 µL of 20% (*w*/*v*) Na_2_CO_3_, and 4 µL of properly diluted sample. Finally, distilled water was added up to 200 µL. After 90 min of stirring on an orbital shaker at RT, the absorbance of each well was read at 734 nm against a blank using a microplate reader. Quantification was performed using gallic acid (GA) as a standard, and results were expressed as mmol of GA equivalent (GAE) per 100 g of fresh weight (FW). The experiments were repeated three times.

#### 2.3.2. Total Proanthocyanidin Content

The total proanthocyanidin content (TPAC) was measured though the Brunswick Laboratories DMAC (BL-DMAC) assay [12], with some modifications [11]. Briefly, 170 µL of the reaction mixture, containing 1 mg/mL of DMAC reagent solubilized in 75% (*v*/*v*) EtOH acidified with 12.5% (*v*/*v*) HCl, were incubated with 60 µL of fruit extract properly diluted in 75% (*v*/*v*) acetone acidified with 0.5% (*v*/*v*) acetic acid. After 10 min of stirring on an orbital shaker at RT, the sample was incubated for 10 min at RT and the absorbance of each well was read at 640 nm against a blank using a microplate reader. The quantification was performed using A2-type Proantocyanidin (PAC-A2) as a standard, and results were expressed as mg of PAC-A2 equivalent (PACE) per 100 g of fresh weight (FW). The experiments were repeated three times.

#### 2.3.3. Total Anthocyanin Content

The total anthocyanin content (TAC) was measured through the pH differential method [11,13], which allows the quantification of anthocyanin compounds excluding potential interference of other colored pigments. Briefly, at 5 µL of each fruit extract, 245 µL of 0.025 M KCl (acidified to pH 1.0 with HCl) or 245 µL of 0.4 M sodium acetate (acidified to pH 4.5 with acetic acid) buffer were added. The absorbances of both solutions were read at 510 nm and 700 nm against the respective blanks. Determination of TAC value was performed using a rearrangement of the Lambert–Beer law, as described by the following equation (Equation (1)):

(1) $$\mathrm{TAC}\;\left(\mathrm{mg}/\mathrm{mL}\right)\;=\;\frac{{\left({\mathrm{Abs}}_{510}-{\mathrm{Abs}}_{700}\right)}_{\mathrm{pH}1}-{\left({\mathrm{Abs}}_{510}-{\mathrm{Abs}}_{700}\right)}_{\mathrm{pH}4.5}\;\times \;\mathrm{MW}\;\times \;1000}{\varepsilon \;\times \;l}\;$$

where: MW is the molecular weight of cyanidin-3-glucoside (449.2 g mol^−1^); ε is the molar extinction coefficient (26,900 mM^−1^ mol^−1^) of cyanidin-3-glucoside; l is the path length (1 cm). Data were expressed as mg of cyanidin -3-glucoside equivalent (CE) per 100 g of FW. The experiments were repeated three times.

#### 2.3.4. Annotation of Bioactive Compounds via HPLC-Orbitrap

Bioactive compounds were putatively identified by high-performance liquid chromatography (HPLC) (Ultimate 3000 HPLC, Thermo Scientific™, Waltham, MA, USA) coupled to an Orbitrap Fusion instrument (Thermo Fisher Scientific Inc., Waltham, MA, USA) equipped with a H-ESI ion source. The separation was achieved using a Luna C18(2) C18 column (150 × 2 mm, 100 Å, 3 µm. The gradient consisted of 0.1% (*v*/*v*) formic acid (solvent A) and pure MetOH supplied with 0.1% (*v*/*v*) formic acid (solvent B). The chromatographic gradient started with 5% (*v*/*v*) solvent B, was maintained for 5 min, and was gradually raised up to 98% (*v*/*v*) for 40 min. Subsequently, the concentration of solvent B was kept constant for 4 min, then the column was re-equilibrated for 6 min [14]. The injection volume for sample was set to 20 µL, whereas the flow rate was at 200 µL/min. In order to identify bioactive compounds in fruit extract, samples were analyzed using an Orbitrap Fusion HRMS in dependent data analysis (DDA) mode. The main tuning parameters adopted for the ESI source were source voltage: 4500 V (+ion mode) 3100 V (−ion mode); capillary temp: 275 °C; sheath gas flow: 35 arb, aux gas flow: 15 arb, sweep gas flow: 0 arb. All mass spectra ranging from 150 to 1500 *m/z* were obtained with resolution of 30,000 (500 *m*/*z* FWHM) in positive- and negative-ionization mode; the threshold for the data-dependent scan triggering was set to 2^5^ counts.

### 2.4. Antioxidant Proprieties

#### 2.4.1. Radical-Scavenging Activity

The radical-scavenging activity was measured via ABTS (Re et al., 1999) and DPPH (Brand-Williams et al., 1995) assays. ABTS^+^ was produced by reacting 7 mM ABTS stock solution with 2.45 mM K_2_S_2_O_8_, allowing the mixture to stand in the dark at RT for 16 h before use. The solution was diluted until it reached a final absorbance of 0.70 at 734 nm. The absorbance at 734 nm was recorded for 5 min after the mixing of 10 µL of extract (or antioxidant standard) with 1 mL ABTS^+^ solution using a spectrophotometer with ethanol as blank. DPPH assay was carried out, adapting the protocol to a spectrophotometric reading using a microplate reader [13]. Briefly, 0.5 mL of 0.1 mM DPPH solution was diluted until it reached a final absorbance of 0.90 at 517 nm. Consequently, 190 µL of the diluted reaction mixture was added to 10 µL of properly diluted fruit extract. After 20 min, the absorbance was read at 517 nm with ethanol as blank. For both assays, the inhibition percentage was calculated using the following equation (Equation (2)):

(2) $$\;\mathrm{IP}\%=\frac{A_{\mathrm{CTR}}-{\;A}_{\mathrm{TEST}}}{A_{\mathrm{CTR}}}\;\times \;100$$

where IP(%) is the percentage of color reduction of the reagent mixture; A_CTR_ is the absorbance of ABTS and DPPH solution at the respective wavelengths (734 nm for ABTS assay or 517 nm for DPPH assay) before the addition of the extract; while A_TEST_ is the absorbance of ABTS and DPPH solution after the addition of the extract read at the respective wavelengths (734 nm for ABTS assay or 517 nm for DPPH assay) at the end of incubation time. Trolox was used as a reference standard, and the antioxidant activity of each assay was expressed as mmol of Trolox equivalent (TE) per 100 g of FW. The experiments were repeated three times.

#### 2.4.2. Metal-Reducing Antioxidant Power

The metal-reducing antioxidant capacity of the fruit extract was evaluated via ferric-reducing antioxidant power (FRAP) assay [15]. Briefly, 300 mM sodium acetate (acidified to pH 3.6 with HCl) was added to 20 mM FeCl_3_ and 10 mM TPTZ in 8:1:1 (*v*/*v*/*v*) ratio. Consequently, 190 µL of the reaction buffer was incubated at 37 °C for 1 h with 10 µL of properly diluted fruit extract. After the incubation time, the absorbance of each well was read at 595 nm against a blank. Trolox was used as a reference standard, and the metal-reducing antioxidant power was expressed as mmol of Trolox equivalent (TE) per 100 g of FW. The experiments were repeated three times.

#### 2.4.3. Cellular Antioxidant Activity assay

Hepatocarcinoma cell line, HepG2, was obtained from American Type Culture Collection (Rockville, MD, USA), cultured in RPMI supplemented with 5% (*v*/*v*) FBS, 2 mM L-glutamine, 50 IU/mL penicillin, and 50 µg/mL streptomycin, and maintained in a humidified atmosphere with 5% CO_2_ at 37 °C [6]. Cells were mostly cultured in 75 cm^2^ culture flasks and were trypsinized using trypsin-EDTA before the confluence was reached. 

The fruit extract was employed for cellular antioxidant activity (CAA) assay [16], which was performed as previously described [13]. Briefly, HepG2 were seeded in 96-well plates at a density equal to 6.0 × 10^4^ cells/well in RPMI medium. After 24 h, the medium was removed and 25 µM DCFH-DA was added in each well along with different concentration of the fruit extract for two hours. In order to ensure that the observed effect depended on the tested sample, the same amount of EtOH contained in the extracts was added to the culture medium of the control cells. However, in all experimental conditions, EtOH never exceeded 0.25% (*v*/*v*). After 2 h of incubation, cells were washed twice with PBS, and then incubated 600 μM ABAP dissolved in HBSS was added. The plates were then placed into a plate-reader thermostat at 37 °C, and emission at 538 nm was measured for one hour, during which an excitation at 485 nm every 5 min was made. Each plate included control and blank wells. Control wells were preincubated with 25 µM DCFH-DA and then treated with 600 μM ABAP. Blank wells were pretreated with 25 µM DCFH-DA and then incubated in HBSS without the oxidant agent. The area under the curve of fluorescence units versus minutes was used to calculate the CAA value for each extract concentration using the following equation (Equation (3)):

(3) $$\mathrm{CAA}=100-\;\left[\frac{\int \mathrm{SA}}{\int \mathrm{CA}}\right]\;\times \;100$$

where CAA is the cellular antioxidant activity; ∫SA is the integrated area under the curve of fluorescence obtained for samples and normalized for blanks; ∫CA is the integrated area under the curve of fluorescence obtained for controls and normalized for blanks.

Finally, the concentration necessary to inhibit 50% of 2′,7′-dichlorofluorescin (DCF) formation (CAA_50_) for each fruit extract was calculated from concentration/response curves using linear regression analysis. Data were expressed as CAA_50_ (mg of FW per mL cell medium). The experiments were repeated three times.

#### 2.4.4. Gene Expression of Antioxidant Enzymes on HepG2 Cells 

To evaluate the expression of antioxidant enzyme genes, HepG2 cells were used, as previously reported [17]. In particular, cells were plated at a density of 5 × 10^5^ cells/well in 12-multiwell plates. After 24 h from the seeding, cells were treated for two hours with the extract using a concentration of about 2-fold CAA_50_ value (1 mg/mL cell medium) in fresh FBS-free RPMI. Then, the culture media were discarded and cells were exposed to 200 µM H_2_O_2_ for 24 h. After the incubation time, total RNA was isolated (RNA-XPress™ Reagent, HiMedia) and reverse-transcribed (OneScript^®^ Reverse Transcriptase, HiMedia, China) in complementary DNA (cDNA) according to the manufacturer’s instructions. The obtained cDNA was then used as a template for quantitative real-time polymerase chain reaction (qRT-PCR), using the BrightGreen 2X qPCR MasterMix-Low ROX (Abm, Richmond, BC, Canada) and QuantStudio™ 3 Real-Time PCR System (Applied Biosystem, Waltham, MA, USA). The primers of both target and reference genes are listed in Table 1. Real-time PCR was performed, as previously described [17], and the relative expression levels of each gene were estimated using the method of Pfaffl [18].

### 2.5. Statystical Analysis

All results were expressed as mean ± standard deviation (SD) of three different technical replicates for each replicate of fruit extract. ANOVA followed by Tukey’s post hoc 255 test was applied with the aim to determine significant differences among the different 256 measurements. A value of *p* ≤ 0.05 was predetermined as the criterion of significance All the statistical analyses were carried out using SPSS Statistics 27 (SPSS, Chicago, IL, USA).

## 3. Results and Discussion

### 3.1. Phytochemical Profile 

In this work, the phytochemical profile of *E. involucrata* fruits was investigated by both spectrophotometric assays and HPLC-Orbitrap analyses. The total contents of polyphenols (TPC), anthocyanins (TAC), and proanthocyanidins (TPAC) were estimated by Folin–Ciocalteu assay, pH differential method, and BL-DMAC assay, respectively (Table 2). 

**Figure 1:**
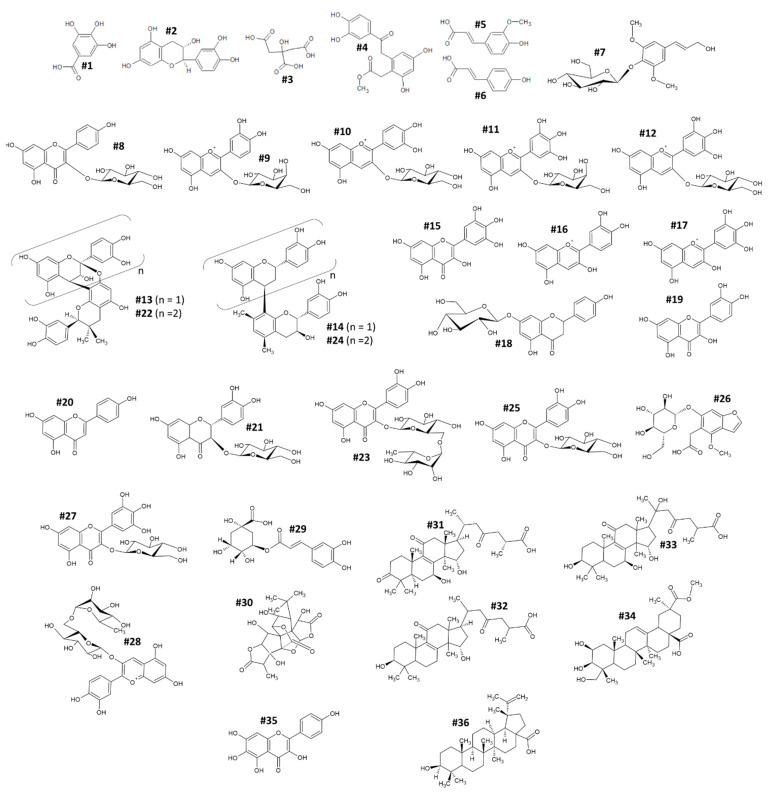
Structural formulas of the bioactive compounds annotated via HPLC-Orbitrap in the extract of Cereja do Rio Grande (*Eugenia involucrata* DC).

On the other hand, HPLC-Orbitrap analysis was used for the identification and profiling of phytochemicals (Figure 1, Table 3).

#### 3.1.1. Total Phenolic, Total Anthocyanin, and Total Proanthocyanidin Content

The comparison of TPC, TAC, and TPAC values for *E. involucrata* fruit extract with previously published data results is difficult. For example, although Girardello and colleagues performed the Folin–Ciocalteu assay to quantify the total content of polyphenols, they reported a TPC value expressed as mg of GAE per mL of extract, without providing any information regarding the volume of solvent used to resuspend the powder after the lyophilization process [10]. In addition, in a recent work published by Infante et al., 18.36 ± 0.66 mg of GAE per gram of dried fruit was recorded as a mean TPC value for different *E. involucrata* fruit extracts, but information concerning the moisture content was not provided by authors. However, considering that in our experimental conditions the flesh fruits had a moisture content of about 90% (*w*/*w*) (data not shown), we can assume that TPC measured by Infante et al. was slightly higher than the value measured in our fruit extract [19].

On the other hand, when comparing the TPC value to those evaluated for other tropical fruits grown in Sicily, such as cherimoya, papaya, and mango [6], *E. involucrata* fruits displayed higher content of polyphenols. However, TPC was lower than those measured in other red-colored fruits, such as grapes [20], cherries, and plums [21].

Anthocyanins are not only the main responsible molecules for the reddish and purple color of various fruits, but are also the important contributors of their health-promoting properties. Indeed, the intake of anthocyanin-enriched fruits was positively correlated to cardiovascular protection, antiobesity and antidiabetic effects, anti-inflammatory, antibacterial, and anticancer activity [22]. The analysis revealed that *E. involucrata* fruits had a good amount of these bioactive pigments (Table 2). Comparing the TAC value with those reported for other red-colored species, *E. involucrata* fruit had fewer anthocyanins than other berries, including blackberries, cranberries, raspberries, and strawberries; however, it is almost comparable with red fruits of species belonging to *Prunus* genus [22,23,24].

PACs are polymers generated by the condensation of several units of flavan-3-ols. Recently, PACs are attracting considerable interest in the nutraceutical field for the treatment of cystitis and other human disorders [12]. Our analysis revealed that *E. involucrata* fruit had a considerable proanthocyanidin content (Table 2). In particular, the TPAC value was comparable to those previously recorded for fruits belonging to *Fragaria*, *Passiflora*, and *Litchi* genera [6]. The presence of PACs in plant foods must not be undervalued. These bioactive compounds, in addition to having a remarkable antioxidant capacity, are limitedly distributed within the plant kingdom. In particular, although most of the red-colored fruits are capable of synthesizing them, some plant genera such as *Plinia*, a genus phylogenetically close to *Eugenia involucrata*, are unable to synthesize PACs due to the lack of reductase enzymes for Leucoanthocyanins and Anthocyanins [12].

#### 3.1.2. Annotation of Phytochemicals

The potential and specific beneficial effects of natural bioactive compounds have increased the interest in identification and profiling of phytochemicals that may compose the different plant raw materials. Moreover, the investigation of these compounds is also becoming important for the proper nutraceutical valorization of fruits, vegetables, and other plant materials that are currently little-known by common consumers. Here, in order to identify the bioactive compounds present in the extract of *E. involucrata* fruit, qualitative studies were assessed by HPLC-Orbitrap instrumentation. This analysis allowed for the annotation of 36 different compounds belonging to different chemical classes (Figure 1). MSI (Metabolomics Standard Initiative) guidelines were followed for the annotation of the following compounds [25].

One organic acid (citric acid (#3)); three phenolic acids (gallic acid (#1), ferulic acid (#5), p-coumaric acid (#6)); five flavan-3-ols (catechin (#2), A-type proanthocyanidin dimer (#13), B-type proanthocyanidin dimer (#14), A-type proanthocyanidin trimer (#22), B-type proanthocyanidin trimer (#24)); three phenolic esters (jaboticabin (#4), syringin (#7), chlorogenic acid (#29); seven flavon-3-ols (astragalin (#8), myricetin (#15), myricetin 3-glucoside (#27), isoquercetin (#25), rutin (#23), quercetin (#19), 6-hydroxykaempferol (#35)); two flavones (apigenin 7-O-glucoside (#18), apigenin (#20)); one flavanonol (dihydroquercetin 3-glucoside (#21)); seven anthocyanins (kuromanine (#9), ideain (#10), myrtillin (#11), empetrin (#12), cyanidin (#16), delphinidin (#17), keracyanin (#28)); benzofuran (picraquassioside a (#26)); and six terpenoids (betulinic acid (#36), ganoderic acid C (#31), ganolucidic acid B (#32), ganoderic acid L (#33), phytolaccagenin (#34), ginkgolide C (#30)). All these compounds are listed in Table 3.

The mass-to-charge ratio (*m/z*) of each compound, along with its Chemical Abstracts Service Identification Number (CAS-ID), retention time (RT), chemical structure, mass fragmentation (MS/MS), and Δppm, are listed in Table 3. The chemical profile is partially in accordance to the literature data [9,19]. In particular, in the studies by Nicacio et al. and Infante et al., HPLC or gas chromatography (GC) coupled to a triple-quadrupole mass spectrometer (QqQ) were used to determine the main bioactive constituents of extracts from *E. involucrata* fruits [9,19]. Their analyses are in accordance with our results, recording the presence of chlorogenic acid (#29), catechin (#2), gallic acid (#1); ferulic acid (#5), and p-coumaric acid (#6), in addition to myricetin (#15) and quercetin (#19). On the other hand, unlike our results, they unexpectedly were not able to find the glycosylated forms of myricetin and quercetin. Indeed, after the aglycone polyphenols are synthesized during the phenylpropanoid and flavonoid pathway in plant cells, they are commonly glycosylated or functionalized with other molecules in order to increase their solubility and availability in the cellular environment [26,27,28]. 

Another recent work aimed at profiling *E. involucrata* fruit extracts was published by Schmidt and colleagues [29]. Here, the authors used an HPLC coupled to a diode array detector (DAD) and to a quadrupole time-of-flight analyzer (QTOF) to compare the phytochemical profile of some berries, including three different Cereja do Rio Grande varieties. The authors detected catechin (#2) and several structurally different PACs: cyanidin, (#16), delphinidin (#17), and quercetin [#18), along with their related glycosylated forms. However, while they detected pelargonidin and kaempferol in all three varieties of Cereja do Rio Grande, no traces of these polyphenols were identified in our extract.

On the other hand, the presence of triterpenoid compounds in *Eugenia involucrata* fruit had not yet been detected. In particular, four tetracyclics (#31, #32, #33, #36), one pentacyclic (#34), and one lactone (#30) were annotated. Triterpene compounds are bioactive compounds that are synthesized in plants through the mevalonate pathway [30]. Currently, more than 1000 different triterpene compounds are known and are used in traditional medicines or dietary supplements because they can exert potential different biological activities, including antinflammatory and hypocholesterol activity [31].

### 3.2. Antioxidant Properties 

#### 3.2.1. Cellular Antioxidant Activity 

The oxidation of membrane lipids and the formation of oxidated lipid products affect membrane function and have been associated with several pathologic conditions characterized by oxidative stress [32]. In order to evaluate the potential antioxidant properties of *E. involucrata* fruit extract, a CAA assay, using a cell-based lipid peroxidation model, was performed [16]. The CAA_50_ of *E. involucrata* fruit extract was 50-fold lower than that determined by Wolfe et al. for 25 common fruits (Table 2), suggesting a high antioxidant potential of this fruit [16]. Moreover, in comparison to rich antioxidant compound fruits, *E. involucrata* fruit extract displayed an antioxidant activity 4-fold higher than wild blueberry and pomegranate [16].

#### 3.2.2. Radical-Scavenging and Metal-Reducing Activities

The antioxidant protection on HepG2 cells may involve different mechanisms, including the direct interaction of fruit antioxidant components with oxidative species. Reactive oxygen (ROS) and nitrogen (RNS) species tend to acquire electrons from other molecules with the aim to stabilize themselves. Then, their stabilization in cells can follow in the oxidation of lipids, proteins, sugars, and nucleic acids, which lose their biological function [33].

In order to evaluate the capability of Cereja do Rio Grande extract to neutralize reactive species by reducing them to more stable species, the total antioxidant capacity was measured by in-solution methods. Currently, different and numerous chemical assays are used for the evaluation of antioxidant activity through specific mechanisms, including scavenging activity against certain types of radicals, metal-reducing power, and metal-chelation property [34]. Since the use of a single method often does not describe the real antioxidant potential of a complex mixture, such as an extract, a combination of methods is preferred.

Here, both radical-scavenging and metal-reducing activities of the extract of *E. involucrata* fruits were evaluated by three in-solution assays. DPPH and ABTS assays evaluate the radical-scavenging activity against hydrophilic radicals, which are transformed into more stable species via the transfer of electrons or hydrogen atoms from redox-active compounds. On the other hand, the FRAP assay evaluates the ability to reduce ferric(III) ions to ferrous(II) ions [15]. The obtained results showed a strong reducing activity of compounds in *E. involucrata* fruit extract (Table 2). In particular, the mean values for antioxidant activity were 2.42 ± 0.06; 1.25 ± 0.05, and 3.64 ± 0.22 mmol TE per 100 g of FW determined by FRAP, DPPH, and ABTS assays, respectively. In particular, the high FRAP value is justified by the presence in the extract of components having meta- and orto-oriented hydroxyl groups that can exert a potential metal-chelating capability [35]. A possible comparison with previously published data turns out to be difficult. Indeed, even if Nicácio and coauthors measured the radical-scavenging activity of *E. involucrata* fruit extracts through DPPH and ABTS assays, they separated pulp from peel without specifying the pulp/peel weight ratio in the whole fruit [9]. Moreover, it was not even possible to compare the DPPH, ABTS, and FRAP values with those obtained in other studies evaluating the antioxidant properties of both edible part (peel + pulp) or whole fruit due to differences in the data expression [19,29] and to the lack of information concerning the methodologies used for concentration process [10]. On the other hand, some studies have evaluated the antioxidant properties of different species belonging to the genus *Eugenia*. The data collected in this work showed that *E. involucrata* fruits have a lower antioxidant activity than other *Eugenia* species. For example, Celli and coauthors evaluated the antioxidant activity of pulp of red and purple *E. uniflora* fruits via DPPH method, reporting a mean value equal to 8.33 and 10.43 mmol TE per 100 g of FW [36]. Moreover, Rufino and colleagues determined the antioxidant activity of 18 tropical fruits grown in Brazil and estimated an ABTS value equal to 182 μmol TE per g of FW for edible portion of *E. pyriformis* fruit extracts [37].

#### 3.2.3. Gene Expression of Antioxidant Enzymes in HepG2 Cells 

The beneficial effects derived from the intake of plant foods have been frequently ascribed to antioxidant properties of their components [38]. The documented capacity of several phytochemicals to affect cellular redox balance, preventing oxidative damage in cells, have been also correlated to the increase in enzymatic antioxidant defenses [11]. Scientific data have shown the ability of different bioactive compounds to cross cell membranes and interact with several biological targets, influencing their conformation and then their function. In this context, the binding of phytochemicals to antioxidant enzymes or proteins involved in their expression can influence their activity and expression levels with important consequences on the cellular redox balance [39].

**Figure 2:**
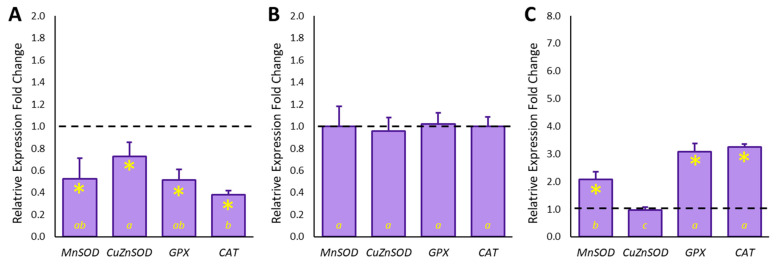
Relative expression of superoxide dismutase (*SOD*), catalase (*CAT*), and glutathione peroxidase (*GPx*) gene in HepG2 cells. (**A**) shows the relative gene expression of cells treated for 24 h with 200 μM H_2_O_2_ in comparison with untreated cells. (**B**) shows the relative gene expression of cells treated for 2 h with *E. involucrata* fruit extract in comparison with untreated cells. (**C**) shows the relative gene expression of cells treated for 2 h with the fruit extract, and then exposed to 200 μM H_2_O_2_ for 24 h in comparison with cells exposed to 200 μM H_2_O_2_ for 24 h. For all experimental conditions, *β-actin* was used as the reference gene. The bars represent the mean ± SD of three qRT-PCR analyses performed in triplicate. Different lowercase letters at the bottom of each bar indicate significant differences in the obtained values, as measured by Tukey’s test (*p* ≤ 0.05). The asterisk (*), when present, indicates statistical differences (*p* ≤ 0.05) between the control condition (dashed line) and the treated condition.

To study the mechanisms involved in the antioxidant protection observed in HepG2 cells, the influence of phytochemicals in *E. involucrata* extract on enzymatic antioxidant defenses was evaluated. In this aim, the gene expressions of four antioxidant enzymes (*MnSOD*, *CuZnSOD*, *CAT*, and *GPx*) were assessed under both normal and pro-oxidative environments, before or after incubation with Cereja do Rio Grande extract (Figure 2). 

SOD is the only enzyme able to neutralize superoxide radical anions, the first reactive species derived from incomplete reduction of molecular oxygen. The neutralization of this radical is crucial because it prevents the production of hydroxyl radical, which is one of the most unstable ROSs [40]. The radical-scavenging activity of SOD is effective only when followed by the action of enzymes, such as CAT and *GPx*, able to neutralize the produced H_2_O_2_. In our experimental model, HepG2 cells expressed all the studied genes. After 24 h of cell exposure to 200 µM H_2_O_2_, a strong downregulation of the target genes was observed, according to previous works [6]. (Figure 2, Panel A). In particular, the strongest downregulation was measured for *CAT* (−2.61-fold) expression. Moreover, pretreatment of unstressed cells with Cereja do Rio Grande extract in a noncytotoxic concentration and 2-fold CAA_50_ value did not affect the basal expression of the observed genes (Figure 2, Panel B). On the other hand, when cells were exposed to *E. involucrata* extract and then to 200 µM H_2_O_2_, a reactivation of the target genes, except *CuZnSOD*, was recorded. In particular, a slight upregulation of *MnSOD* (+2.1-fold) and a strong upregulation of *GPx* (+3.1-fold) and *CAT* (+3.3-fold) genes were observed (Figure 2, Panel C). The concomitant activation of the superoxide radical anion scavenger enzyme and of CAT and *GPx*, involved in H_2_O_2_ scavenging, suggests that the extract components are able to effectively coordinate the antioxidant response in cells.

## 4. Conclusions

The results obtained in this work provide for the first time a complete qualitative phytochemical profile and a summary of antioxidant properties of Cereja do Rio Grande. In particular, collected data demonstrated that hydroalcoholic extract of Cereja do Rio Grande is a rich source of bioactive compounds able to prevent oxidative damage, making cells more resilient to oxidative stimuli. In addition, the obtained data indicate that observed antioxidant protection implicates both redox-active properties of extract components and their capability to increase the enzymatic antioxidant defenses. Finally, the results indicate that *E. involucrata* is a fruit with potential beneficial effects on human health and may find a use as raw material for the nutraceutical, pharmaceutical, and cosmetic industries.

## Figures and Tables

**Table 1:** PCR primer sequences used for quantitative real-time polymerase chain reaction (qRT-PCR) analysis.

Genes		Primer Sequences	Accession
*CuZnSOD*	F	5′-ACGGTGGGCCAAAGGATGAA-3′	AC026776.4
	R	5′-TCATGGACCACCAGTGTGCG-3′	
*MnSOD*	F	5′-AGAAGCACAGCCTCCCCGAC-3′	NM_000636.4
	R	5′-GGCCAACGCCTCCTGGTACT-3′	
*GPx*	F	5′-TCGGTGTATGCCTTCTCGGC-3′	NM_000581.4
	R	5′-CCGCTGCAGCTCGTTCATCT-3′	
*CAT*	F	5′-CCAACAGCTTTGGTGCTCCG-3′	NM_001752.4
	R	5′-GGCCGGCAATGTTCTCACAC-3′	
*β-Actin*	F	5′-CGGGAAATCGTGCGTGACAT-3′	NM_001101.5
	R	5′-GGACTCCATGCCCAGGAAGG-3′	

F: forward primer; R: reverse primer; SOD: superoxide dismutase; *GPx*: glutathione peroxidase; CAT: catalase.

**Table 2:** Total content of polyphenols, proanthocyanidins, and anthocyanins of *Eugenia involucrata* fruit extract along with its radical-scavenging (DPPH and ABTS), metal-reducing (FRAP), and cellular antioxidant activity.

Assay	Content	Unit of Measurement
**TPC**	136.83 ± 9.5	mg GAE per 100 g of FW
**TPAC**	59.27 ± 0.32	mg PACE per 100 g of FW
**TAC**	75.26 ± 0.74	mg CE per 100 g of FW
**DPPH**	1.25 ± 0.05	mmol TE per 100 g of FW
**ABTS**	3.64 ± 0.22	mmol TE per 100 g of FW
**FRAP**	2.42 ± 0.06	mmol TE per 100 g of FW
**CAA50**	0.530 ± 0.045	mg of FW per mL cell medium

GAE: gallic acid equivalent; FW: fresh weight; PACE: proanthocyanidin A2 equivalent; CE: cyanidin-3-glucoside equivalent; TE: Trolox equivalent; FRAP: ferric-reducing antioxidant power; TPC: total polyphenol content; TPAC: total proanthocyanidin content; TAC: total anthocyanin content; CAA: cellular antioxidant activity.

**Table 3:** Bioactive compounds annotated in *Eugenia involucrata* fruit extract. The columns report CAS-ID, retention time (RT), mode of analysis (−/+), name and structural formula, MSI annotation level, charged molecular weight (*m/z*), observed mass fragmentation, and mass accuracy shift in ppm (Δppm).

#	CAS-ID	RT	Mode	Chemical Name	MSI Level	Chemical Structure	*m*/*z*	MS/MS Fragmentation	Δ_ppm_
**Organic Acid**									
**3**	77-92-9	4.49	−	Citric Acid	2	C_6_H_8_O_7_	191.0202	111.0094 (100); 173.0096 (12)	+2.011
**Phenolic Acids**									
**1**	149-91-7	4.31	−	Gallic Acid	3	C_7_H_6_O_5_	303.1902		+0.115
**5**	537-98-4	4.79	−	Ferulic Acid	2	C_10_H_10_O_4_	193.1842	134.0094 (100); 149.2314 (50)	+0.128
**6**	501-98-4	4.81	−	4-Coumaric Acid	2	C_9_H_8_O_3_	163.0473	145.9019 (100); 162.9058 (10)	+0.265
**Flavan-3-ols**									
**2**	7295-85-4	4.36	−	Catechin	3	C_15_H_14_O_6_	289.2712		+0.125
**13**	41743-41-3	13.58	−	A-type Proanthocyanidin Dimer	2	C_30_H_24_O_12_	575.5012	405.1230 (100); 421.3257 (54); 289.2321 (10)	−4.412
**14**	20315-25-7	13.95	−	B-type Proanthocyanidin Dimer	2	C_30_H_26_O_12_	577.5121	407.0778 (100); 451.1039 (38); 423.0715 (13)	−3.214
**22**	N/A	15.38	−	A-type Proanthocyanidin Trimer	2	C_45_H_34_O_18_	861.3732	575.2120 (100); 449.2147 (45); 289.2320 (10)	+0.369
**24**	N/A	15.58	−	B-type Proanthocyanidin Trimer	2	C_45_H_38_O_18_	865.2425	577.5112 (100); 449.1485 (32); 289.2320 (10)	+0.754
**Polyphenolic Esthers**									
**4**	N/A	4.77	−	Jaboticabin	3	C_16_H_14_O_8_	333.0591		−2.718
**7**	118-34-3	4.81	−	Syringin	3	C_17_H_24_O_9_	371.1331		−4.461
**29**	327-97-9	17.52	−	Chlorogenic Acid	3	C_16_H_18_O_9_	353.0952		−0.221
**Flavonols**									
**8**	480-10-4	4.91	−	Astragalin	2	C_21_H_20_O_11_	447.0934	287.0553 (100)	−0.326
**15**	529-44-2	14.21	−/+	Myricetin	2	C_15_H_10_O_8_	317.2371	109.0262 (100)	−2.615
**27**	19833-12-6	16.78	−/+	Myricetin 3-glucoside	2	C_21_H_20_O_13_	480.3782	316.0248 (100); 271.0218 (50)	−0.874
**25**	482-35-9	15.65	+	Isoquercetin	2	C_21_H_20_O_12_	465.1029	303.0503 (100)	+0.468
**23**	153-18-4	15.42	−/+	Rutin	2	C_27_H_30_O_16_	611.1613	303.0503 (100); 465.1028 (37)	+0.181
**19**	117-39-5	15.27	−/+	Quercetin	2	C_15_H_10_O_7_	301.0426	178.9985 (100)	−1.254
**35**	4324-55-4	23.95	−	6-Hydroxykaempferol	3	C_15_H_10_O_7_	301.0381		−0.126
**Flavones**									
**18**	578-74-5	15.22	−/+	Cosmosiin	2	C_21_H_20_O_10_	433.1135	271.0605 (100)	−1.041
**20**	520-36-5	15.29	−/+	Versulin	3	C_15_H_10_O_5_	271.0602		+0.406
**Flavanonol**									
**21**	27297-45-6	15.34	−	Taxifolin	2	C_21_H_22_O_12_	467.1187	305.0662 (100)	+0.209
**Anthocyanins**									
**9**	7084-24-4	7.53	+	Kuromanine	2	C_21_H_21_O_11_^+^	449.1084	287.0555 (100)	+1.250
**10**	27661-36-5	8.42	+	Ideain	2	C_21_H_21_O_11_^+^	449.1084	287.0555 (100)	+1.250
**11**	50986-17-9	12.61	+	Myrtillin	2	C_21_H_21_O_12_^+^	465.1029	303.0504 (100)	−0.776
**12**	50986-17-9	13.32	+	Empetrin	2	C_21_H_21_O_12_^+^	465.1029	303.0504 (100)	−0.776
**16**	13306-05-3	14.25	+	Cyanidin	3	C_15_H_11_O_6_^+^	287.0554		−2.617
**17**	528-53-0	15.21	+	Delphinidin	3	C_15_H_11_O_7_^+^	303.0504		−1.901
**28**	28338-59-2	17.02	+	Keracyanin	2	C_27_H_31_O_15_^+^	595.1663	287.0556 (100); 449.1081 (41)	+0.143
**Benzofuran**									
**26**	169312-28-1	15.78	+	Picraquassioside A	2	C_18_H_22_O_10_	399.1291	237.0702 (100)	−0.079
**Terpenoids**									
**36**	472-15-1	24.02	−/+	Betulinic acid	2	C_30_H_48_O_3_	457.3679	421.3468 (100); 439.3572 (97)	+0.717
**31**	81907-62-2	18.39	−/+	Ganoderic Acid C	2	C_30_H_46_O_7_	519.3315	473.3259 (100); 455.3154 (75); 483.3102 (62)	−0.289
**32**	98683-75-1	19.21	−/+	Ganolucidic Acid B	3	C_30_H_44_O_6_	501.3321		+0.627
**33**	102607-24-9	23.14	−/+	Ganoderic Acid L	2	C_30_H_46_O_8_	535.3268	517.3159 (100); 499.3053 (60); 489.3212 (20)	+0.551
**34**	1802-12-6	23.25	−/+	Phytolaccagenin	2	C_31_H_48_O_7_	533.3475	191.3001 (100); 179.2154 (22)	−0.897
**30**	15291-76-6	18.15	−/+	Ginkgolide C	2	C_20_H_24_O_11_	441.1395	237.0762 (100)	+1.025

## Data Availability

Not applicable.
